# Stability criteria on delay-dependent robust stability for uncertain neutral stochastic nonlinear systems with time-delay

**DOI:** 10.1186/s13660-018-1886-5

**Published:** 2018-10-26

**Authors:** Tengyu Ma, Longsuo Li

**Affiliations:** 1 Department of Mathematics, Harbin Institute of Technology, Harbin, China; 2 Department of Mathematics, Qiqihar University, Qiqihar, China

**Keywords:** Uncertain neutral stochastic systems, One-sided Lipschitz condition, Lyapunov–Krasovskii functional, Linear matrix inequality (LMI)

## Abstract

This work mainly studies the robust stability analysis and design of a controller for uncertain neutral stochastic nonlinear systems with time-delay. Using a modified Lyapunov–Krasovskii functional and the free-weighting matrices technique, we establish some new delay-dependent criteria in terms of linear matrix inequality (LMI). The innovative point of this work is that we generalize the robust stability analysis of nonlinear stochastic time-delay systems to the uncertain neutral stochastic systems. Due to the added derivative term of time-delay, the proposed scheme can be applied more widely. Finally, numerical examples are provided to validate the derived results.

## Introduction

Time-delay systems are widely used to model concrete systems in engineering sciences, such as biology, chemistry, mechanics [1–3]. Time-delay systems, with the rate of current state affected by past state, are negative for the analysis and design of control systems since they may be responsible for performance degradation and instability [4, 5]. There are many valuable results about stability conditions for time-delay systems [6–9]. Generally, the delay-dependent stability condition is less conservative than the delay-independent one. Thus, pursuing the delay-dependent stability condition motivates the present study [10–14].

Over the past years, the Brownian motion phenomenon has been common in biology, economics, and engineering applications. Considerable attention has been devoted to stochastic systems governed by Itô stochastic differential equations, where the noises are described by Brownian motion [15, 16]. A large number of works that focused on stochastic time-delay systems have been published; see, for example, [17–20]. Wang et al. in [19] considered the problems of non-fragile robust stochastic stabilization and robust $H_{\infty }$ control for uncertain stochastic nonlinear time-delay systems. Both the robust stability analysis and non-fragile robust control for a class of uncertain stochastic nonlinear time-delay systems that satisfy a one-sided Lipschitz condition were investigated in [17]. Based on the stochastic Lyapunov–Krasovskii stability approach, the problem of stochastic stability analysis was investigated for $H_{\infty }$ control of uncertain stochastic Markovian jump systems (SMJSs) with mixed time-varying delays [18]; meanwhile, some delay-dependent sufficient conditions on the stochastic stability and *γ*-disturbance attenuation were presented.

The neutral stochastic systems can effectively model a class of physical dynamical systems, since the mathematical models of them include the time-delays of state and its derivative. These models have received considerable attention recently [21–25]. In fact, neutral stochastic systems are applied widely in automatic control, aircraft stabilization, lossless transmission lines, and system of turbojet engine [26–28]. Both the stability analysis and synthesis of neutral stochastic systems have been extensively studied [29–36]. By using the generalized integral inequality and the nonnegative local martingale convergence theorem, the authors in [30] investigated the exponential stability and the almost sure exponential stability of neutral stochastic delay systems (NSDSs) with Markovian switching. The authors in [33] constructed a new sliding surface functional and considered the $H_{\infty }$ sliding mode control (SMC) for uncertain neutral stochastic systems with Markovian jumping parameters and time-varying delays. Using a delayed output-feedback control method, Karimi et al. in [36] designed a controller, which guarantees $H_{\infty }$ synchronization of the second-order neutral master and slave systems.

On the other hand, a useful one-sided Lipschitz condition was developed in [37]. Since the nonlinear part satisfying this condition can make positive contributions to the stability of systems, we can easily solve the observer design problem for nonlinear systems [17, 38–40]. Inspired by the above works, we investigate the robust stability of neutral stochastic time-delay nonlinear systems with one-sided Lipschitz condition.

In this paper, we propose a class of uncertain neutral stochastic nonlinear systems. Since the systems have a derivative term of time-delay of state, they can be used in lots of fields. We investigate the nonlinear function with both one-sided Lipschitz condition and a quadratic inner-bounded condition. Firstly, a delay-dependent sufficient condition is proposed by constructing an appropriate Lyapunov–Krasovskii functional based on the free-weighting matrices method. Secondly, we construct a memory-less non-fragile state-feedback controller to guarantee asymptotical stability of the closed-loop systems. Finally, we present some numerical examples to illustrate the advantages and effectiveness of our results and find that the proposed method is less conservative.

The organization of this paper is given as follows. In the next section, we recall some notations, lemmas, and definitions of stochastic differential equations. In Sect. 3, the main problems are formulated. In Sect. 4, we give two delay-dependent sufficient conditions for uncertain neutral stochastic nonlinear time-delay systems. In Sect. 5, we design a memory-less non-fragile state-feedback controller to guarantee that the closed-loop systems are asymptotically stable, and in Sect. 6, we present two numerical examples to demonstrate the validity of the mentioned method. The last section contains a conclusion.

## Notations and preliminaries

In this section, we introduce some basic concepts, properties, and notations. These basic facts can be found in any introductory book on stochastic differential equations; see, for example, [41–43].

Throughout this paper, let $(\varOmega ,\mathcal{F},\mathcal{P})$ be a complete probability space with a filtration $\{\mathcal{F}_{t}\}_{t\geq 0}$ satisfying the usual conditions (i.e., the filtration contains all $\mathcal{P}$-null sets and is right continuous). $B(t)$ is a one-dimensional Brownian motion defined on the probability space adapted to the filtration. $\mathbb{R}^{n}$ and $\mathbb{R}^{m\times n}$ denote the *n*-dimensional Euclidean space and the set of all $m\times n$ real matrices, respectively. $\Vert \cdot \Vert _{2}$ stands for the usual $L_{2}[0,\infty )$ norm. The inner product of vectors *x* and *y* in $\mathbb{R}^{n}$ is denoted by $< x,y>$ or $x^{T}y$. Let $C^{2,1}(\mathbb{R}^{n}\times \mathbb{R}_{+}; \mathbb{R}_{+})$ denote the family of all real-valued functions $V(x(t),t)$ defined on $\mathbb{R}^{n}\times \mathbb{R}_{+}$ such that they are continuously twice differentiable in *x* and once in *t*. $C([-\tau ,0]; \mathbb{R}^{n})$ denotes the space of all continuous $\mathbb{R}^{n}$-valued functions *φ* defined on $[-\tau , 0]$ with a norm $\Vert \varphi \Vert =$ sup$_{-\tau \leq \theta \leq 0}\vert \varphi (\theta ) \vert $. The notation $P > 0$ means that *P* is real symmetric and positive definite; the asterisk “∗” denotes a matrix that can be inferred by symmetry and the superscript “*T*” represents the transpose of a matrix or a vector. In a matrix, $(i,j)$ denotes an $(i,j)$-block element of the matrix. The notation $\mathbf{E}\{\cdot \}$ represents the mathematical expectation operator. *I* denotes the identity matrix of compatible dimension.

### Definition 2.1

([17])

The nonlinear function $f(x,y)$ is said to be one-sided Lipschitz if there exist $\alpha _{1}, \alpha _{2} \in \mathbb{R}$ such that

1 $$ \bigl\langle f(x,y),x \bigr\rangle \leq \alpha _{1}x^{T} x+\alpha _{2}y^{T} y $$

for $\forall x, y \in \mathbb{R}^{n}$, where constants $\alpha _{1}$ and $\alpha _{2}$ are positive, zero, or even negative, and they are called one-sided Lipschitz constants for $f(x,y)$ with respect to *x* and *y*.

### Definition 2.2

([17])

The nonlinear function $f(x,y)$ is called quadratic inner-bounded in the region *C* if, for any $x,y \in C$, there exist constants $\beta _{1}$, $\beta _{2}$, and *γ* such that

2 $$ f(x,y)^{T}f(x,y) \leq \beta _{1}x^{T} x+\beta _{2}y^{T} y+\gamma \bigl\langle x ,f(x,y) \bigr\rangle . $$

### Lemma 2.1

(Schur complement [43])

*For a given symmetric matrix*

$$ S= \begin{bmatrix} S_{11}&S_{12}\\ S_{12}^{T}&S_{22} \end{bmatrix}, $$

*the following conditions are equivalent*:

1. $S<0$,
2. $S_{11}<0$, $S_{22}-S_{12}^{T}S_{11}^{-1}S_{12}<0$,
3. $S_{22}<0$, $S_{11}-S_{12}S_{22}^{-1}S_{12}^{T}<0$.

### Lemma 2.2

([44])

*Let* $E \in \mathbb{R}^{n}$, $G \in \mathbb{R}^{n}$, *and* $\varepsilon > 0$. *Then we have*

$$ E^{T}G+G^{T}E \leq \varepsilon G^{T}G + \varepsilon ^{-1} E^{T}E. $$

### Lemma 2.3

(S-procedure [45])

*Denote the set* $\mathcal{Z}=\{z\}$, *and let* $\mathcal{F}(z)$, $y_{1}(z)$, $y_{1}(z)$, …, $y_{k}(z)$ *be some functions or functionals*. *Further define the domain* $\mathcal{D}$ *as follows*:

$$ \mathcal{D}= \bigl\{ z\in \mathcal{Z}: y_{1}(z)\geq 0, y_{2}(z)\geq 0,\ldots ,y_{k}(z)\geq 0 \bigr\} , $$

*and the two following conditions*:

1. $\mathcal{F}(z)>0, \forall z\in \mathcal{D}$,
2. ∃ $\varepsilon _{1}\geq 0, \varepsilon _{2}\geq 0,\ldots ,\varepsilon _{k}\geq 0$ *such that*
  $$ \mathcal{S}(\varepsilon ,z)=\mathcal{F}(z)-\sum_{i=1}^{k} \varepsilon _{i} y_{i}(z)>0, \quad \forall z\in \mathcal{Z}. $$

*Then* (2) *implies* (1). *The procedure of replacing* (1) *by* (2) *is called the S*-*procedure*.

## Problem formulation

Consider the following uncertain neutral stochastic time-delay system described in Itô’s form:

3 $$ \textstyle\begin{cases} \mathrm{d} [x(t)-Cx (t-\tau (t) ) ] \\ \quad = [ (A+\Delta A(t) )x(t)+ (A_{\tau }+\Delta A_{\tau }(t) )x (t-\tau (t) ) \\ \quad \quad {}+f(x(t),x (t-\tau (t) )+Uu(t) ]\,\mathrm{d}t \\ \quad \quad {}+ [ (H+\Delta H(t) )x(t)+ (H_{\tau }+ \Delta H_{\tau }(t) )x (t-\tau (t) ) ]\,\mathrm{d}B(t), \\ x(t)= \phi (t),\quad t\in [-\tau ,0], \end{cases} $$

where $x(t)\in \mathbb{R}^{n}$ is the state vector, $u(t)\in \mathbb{R}^{p}$ is the control input, and $\tau (t)$ is the unknown time-varying delay satisfying $0\leq \tau (t)< \tau $ and $\dot{\tau } (t)\leq d$ with real constants *τ*, *d*. $f(x(t),x(t-\tau (t)))\in \mathbb{R}^{n}$ is a nonlinear function with respect to the state $x(t)$ and the delayed state $x(t-\tau (t))$, $f(0,0)=0$. $\phi (t)\in C([-\tau ,0]; \mathbb{R}^{n})$ is a continuous vector-valued initial function, and $B(t)$ is a one-dimensional Brownian motion satisfying

$$ \mathbf{E} \bigl\{ \mathrm{d}B(t) \bigr\} =0, \qquad \mathbf{E} \bigl\{ \mathrm{d}B(t) \bigr\} ^{2}=\mathrm{d}t, $$

in which $A, A_{\tau }, C, H, H_{\tau } \in \mathbb{R}^{n\times n}$, and $U \in \mathbb{R}^{n\times p}$ are known real constant matrices of appropriate dimensions. Moreover, $\Delta A(t)$, $\Delta A_{\tau }(t)$, $\Delta H(t)$, and $\Delta H_{\tau }(t)$ are unknown matrices representing time-varying parameter uncertainties and are assumed to be of the form

4 $$ \begin{bmatrix} \Delta A(t)& \Delta A_{\tau }(t) \\ \Delta H(t)& \Delta H_{\tau }(t) \end{bmatrix}= \begin{bmatrix} E_{1} \\ E_{2} \end{bmatrix} F(t) \bigl[G_{1}\quad G_{2}\bigr], $$

where $E_{1}$, $E_{2}$, $G_{1}$, and$G_{2}$ are known real constant matrices and $F(t)$ is an unknown time-varying matrix function satisfying

5 $$ F^{T}(t)F(t)\leq I,\quad \forall t\in\mathbb{R}^{+}. $$

The parameter uncertainties $\Delta A(t)$, $\Delta A_{\tau }(t)$, $\Delta H(t)$, and $\Delta H_{\tau }(t)$ are said to be admissible if both (4) and (5) hold [17].

## Robust stability analysis

Let

$$\begin{aligned}& h(t) = F(t) \bigl[G_{1} x(t)+G_{2} x \bigl(t-\tau (t) \bigr) \bigr], \\& h_{1}(t) = Ax(t)+A_{\tau }x \bigl(t-\tau (t) \bigr)+f \bigl(x(t),x \bigl(t-\tau (t) \bigr) \bigr)+E_{1}h(t)+Uu(t). \end{aligned}$$

System (3) can be rewritten as follows:

6 $$ \textstyle\begin{cases} \mathrm{d} [x(t)-Cx (t-\tau (t) ) ] = h_{1}(t)\,\mathrm{d}t+ [Hx(t)+H_{\tau }x (t-\tau (t) )+E_{2}h(t) ]\,\mathrm{d}B(t), \\ h(t)^{T}h(t) \leq [G_{1} x(t)+G_{2} x (t-\tau (t) ) ]^{T} [G_{1} x(t)+G_{2} x (t-\tau (t) ) ]. \end{cases} $$

We will consider the problem of robust stability for time-varying delay system (6) with $u(t)=0$.

### Theorem 4.1

*Consider the neutral stochastic time*-*delay system* (6) *with* $u(t)=0$. *The nonlinear function* $f(x(t),x(t-\tau (t)))$ *satisfies* (1) *and* (2). *For given scalars* *τ* *and* *d*, *if there exist matrices* $P>0$, $W_{1}>0$, $W_{2}>0$, $R>0$, $M_{i}>0$ ($i=1,\ldots, 5$), *and* $N_{j}$ ($j=1,\ldots, 4$) *of appropriate dimensions and positive scalars* $\varepsilon _{1}$, $\varepsilon _{2}$, $\varepsilon _{3}$ *satisfying the following LMIs*:

7 $$ \begin{bmatrix} \bar{\varOmega }_{11}& \bar{\varOmega }_{12}& 0 & N_{2}^{T}& 0& \bar{\varOmega }_{16}&\bar{\varOmega }_{17}&A^{T}R&\bar{M}_{1} \\ \ast & \bar{\varOmega }_{22}& N_{3}^{T}&\bar{\varOmega }_{24} &N_{4}^{T}& -C^{T}P&\bar{\varOmega }_{27}&A^{T}_{\tau }R&\bar{M_{2}}\\ \ast & \ast & \bar{\varOmega }_{33}& \bar{\varOmega }_{34}& \bar{\varOmega }_{35}& 0&0&0&\bar{M}_{3}\\ \ast & \ast & \ast & \bar{\varOmega }_{44}& \bar{\varOmega }_{45}&0&0&0&\bar{M}_{4}\\ \ast & \ast & \ast & \ast &\bar{\varOmega }_{55}& 0&0&0&\bar{M}_{5}\\ \ast & \ast & \ast & \ast & \ast & -\varepsilon _{3} I&0&R&0\\ \ast & \ast & \ast & \ast & \ast &\ast &\bar{\varOmega }_{77}&E_{1}^{T} R&0\\ \ast & \ast & \ast & \ast & \ast &\ast &\ast &-\tau ^{-1}R &0\\ \ast & \ast & \ast & \ast & \ast &\ast &\ast &\ast &-\bar{M} \end{bmatrix} < 0, $$

*where*

8 $$\begin{aligned}& \bar{ \varOmega }_{11}=PA+A^{T}P+H^{T}PH+W_{1}+W_{2}+ \varepsilon _{1}G_{1}^{T}G_{1} + \varepsilon _{2}\alpha _{1}I+\varepsilon _{3} \beta _{1}I, \\& \bar{\varOmega }_{12}=PA_{\tau }-A^{T}PC+H^{T}PH_{\tau } +N_{1}^{T}+\varepsilon _{1}G_{1}^{T}G_{2}, \\& \bar{\varOmega }_{16}=P-\frac{1}{2}\varepsilon _{2}I+ \frac{1}{2}\varepsilon _{3}\gamma I,\qquad \bar{\varOmega }_{17}= PE_{1}+H^{T}PE_{2}, \\& \bar{\varOmega }_{22}=-C^{T}PA_{\tau }-A_{\tau }^{T}PC+H_{\tau }^{T}PH_{\tau }-(1-d)W_{1} \\& \hphantom{\bar{\varOmega }_{22}=}{}-N_{1}(C+I)-(C+I)^{T}N_{1}^{T}+ \varepsilon _{1}G_{2}^{T}G_{2}+ \varepsilon _{2}\alpha _{2}I+\varepsilon _{3} \beta _{2}I, \\& \bar{\varOmega }_{24}=N_{1}C-C^{T}N_{2}^{T}-N_{2}^{T}, \qquad\bar{\varOmega }_{27}=-C^{T}PE_{1}+H_{\tau }^{T}PE_{2} \\& \bar{\varOmega }_{33}=-W_{2} -N_{3}-N_{3}^{T}, \qquad \bar{\varOmega }_{34}=-N_{3}C,\qquad \bar{\varOmega }_{35}=N_{3}C-N_{4}^{T}, \\& \bar{\varOmega }_{44}=N_{2}C+C^{T}N_{2}^{T},\qquad \bar{\varOmega }_{45}=-C^{T} N_{4}^{T}, \qquad \bar{\varOmega }_{55}= N_{4}C+C^{T} N_{4}^{T}, \\& \bar{\varOmega }_{77}=E_{2}^{T}PE_{2} - \varepsilon _{1} I,\qquad \bar{ M}= \operatorname{diag} \bigl\{ \tau ^{-1}M_{1}, \tau ^{-1}M_{2},\tau ^{-1}M_{3},\tau ^{-1}M_{4},\tau ^{-1}M_{5} \bigr\} , \\& \bar{ M}_{1}=[M_{1},0,0,0,0], \qquad \bar{ M}_{2}= [0,M_{2},0,0,0],\qquad \bar{ M}_{3}= [0,0,M_{3},0,0], \\& \bar{ M}_{4}= [0,0,0,M_{4},0],\qquad \bar{ M}_{5}= [0,0,0,0,M_{5}], \\& \varLambda = \begin{bmatrix} -M_{1}& 0 & 0& 0& 0& 0 \\ \ast & -M_{2}& 0 & 0& 0& 0\\ \ast & \ast & -M_{3}& 0 & 0& -N_{3}\\ \ast & \ast & \ast & -M_{4}& 0 & 0\\ \ast & \ast & \ast & \ast & -M_{5}&-N_{4}\\ \ast & \ast & \ast & \ast & \ast & -R \end{bmatrix} < 0, \end{aligned}$$

9 $$\begin{aligned}& \varPi = \begin{bmatrix} -M_{1}& 0 & 0& 0& 0& 0 \\ \ast & -M_{2}& 0 & 0& 0& -N_{1}\\ \ast & \ast & -M_{3}& 0 & 0& 0\\ \ast & \ast & \ast & -M_{4}& 0 & -N_{2}\\ \ast & \ast & \ast & \ast & -M_{5}& 0 \\ \ast & \ast & \ast & \ast & \ast & -R \end{bmatrix} < 0, \end{aligned}$$

*then the null solution of the stochastic time*-*delay system* (6) *is asymptotically stable in the mean square*.

### Proof

Choose the following Lyapunov–Krasovskii functional:

10 $$ V \bigl(x(t),t \bigr)=\sum_{i=1}^{4}V_{i}, $$

where

$$\begin{aligned}& V_{1}= \bigl[x(t)-Cx \bigl(t-\tau (t) \bigr) \bigr]^{T} P \bigl[x(t)-Cx \bigl(t-\tau (t) \bigr) \bigr], \qquad V_{2}= \int _{t-\tau (t)}^{t} x^{T}(s) W_{1} x(s)\,\mathrm{d}s, \\& V_{3}= \int _{t-\tau }^{t} x^{T}(s) W_{2} x(s)\,\mathrm{d}s, \qquad V_{4}= \int _{-\tau }^{0} \int _{t+\theta }^{t}h_{1}^{T}(s) R h_{1}(s) \,\mathrm{d}s\,\mathrm{d}\theta . \end{aligned}$$

Using Itô’s formula [41], we obtain the stochastic differential of $V(x(t),t)$ as follows:

11 $$\begin{aligned} &\mathrm{d} {V} \bigl(x(t),t \bigr) \\ &\quad = \mathcal{L}V \bigl(x(t),t \bigr)\,\mathrm{d}t+2 \bigl[x(t)-Cx \bigl(t-\tau (t) \bigr) \bigr]^{T} P \bigl[Hx(t)+H_{\tau }x \bigl(t-\tau (t) \bigr)+E_{2}h(t) \bigr]\,\mathrm{d}B(t), \\ &\quad = \biggl\{ 2 \bigl[x(t)-Cx \bigl(t-\tau (t) \bigr) \bigr]^{T} P \bigl[Ax(t)+A_{\tau }x \bigl(t-\tau (t) \bigr)+f \bigl(x(t),x \bigl(t-\tau (t) \bigr) \bigr)+E_{1}h(t) \bigr] \\ &\quad \quad {} + \bigl[Hx(t)+H_{\tau }x \bigl(t-\tau (t) \bigr)+E_{2}h(t) \bigr]^{T} P \bigl[Hx(t)+H_{\tau }x \bigl(t-\tau (t) \bigr)+E_{2}h(t) \bigr] \\ &\quad \quad {}+x^{T}(t) W_{1} x(t)-(1-\dot{\tau }(t)x^{T} \bigl(t-\tau (t) \bigr) W_{1} x \bigl(t-\tau (t) \bigr) \\ &\quad \quad {}+x^{T}(t) W_{2} x(t)-x^{T}(t-\tau ) W_{2} x(t-\tau ) \\ &\quad \quad {}+\tau h_{1}^{T}(t) R h_{1}(t)- \int _{t-\tau }^{t} h_{1}^{T}(s) R h_{1}(s) \mathrm{d}s \biggr\} \,\mathrm{d}t \\ &\quad \quad {}+ \bigl\{ 2 \bigl[x(t)-Cx \bigl(t-\tau (t) \bigr) \bigr]^{T} P \bigl[Hx(t)+H_{\tau }x \bigl(t-\tau (t) \bigr)+E_{2}h(t) \bigr] \bigr\} \,\mathrm{d}B(t). \end{aligned}$$

Taking the expectation of both sides of (11), we have

12 $$ \mathbf{E}\,\mathrm{d}V \bigl(x(t),t \bigr)=\mathbf{E}\mathcal{L}V \bigl(x(t),t \bigr)\,\mathrm{d}t. $$

Set

$$\begin{aligned}& x_{\tau } \bigl(t-\tau (t) \bigr) \equiv x \bigl(t-\tau (t)-\tau \bigl(t- \tau (t) \bigr) \bigr), \\& x_{\tau }(t-\tau ) \equiv x \bigl(t-\tau -\tau (t-\tau ) \bigr). \end{aligned}$$

Using the Newton–Leibniz formula and the free-weighting matrices technique, we can derive the following equations:

13 $$\begin{aligned}& 2 \bigl[x^{T} \bigl(t-\tau (t) \bigr) N_{1}+x^{T}_{\tau } \bigl(t-\tau (t) \bigr) N_{2} \bigr] \biggl[x(t)-Cx \bigl(t-\tau (t) \bigr)-x \bigl(t- \tau (t) \bigr)+Cx_{\tau } \bigl(t-\tau (t) \bigr) \\& \quad {}-\int _{t-\tau (t) }^{t} h_{1}(s)\,\mathrm{d}s- \int _{t-\tau (t) }^{t} \bigl(Hx(s)+H_{\tau }x \bigl(s- \tau (s) \bigr)+E_{2}h(s) \bigr)\,\mathrm{d}B(s) \biggr]=0, \end{aligned}$$

14 $$\begin{aligned}& 2 \bigl[x^{T}(t-\tau ) N_{3}+x^{T}_{\tau }(t-\tau ) N_{4} \bigr] \biggl[ x \bigl(t-\tau (t) \bigr)-Cx_{\tau } \bigl(t-\tau (t) \bigr)-x(t-\tau )+Cx_{\tau }(t-\tau ) \\& \quad {}-\int _{t-\tau }^{t-\tau (t)}h_{1}(s)\,\mathrm{d}s- \int _{t-\tau }^{t-\tau (t)} \bigl(Hx(s)+H_{\tau }x \bigl(s- \tau (s) \bigr)+E_{2}h(s) \bigr)\,\mathrm{d}B(s) \biggr]=0, \end{aligned}$$

where $N_{j}$ ($j=1,\ldots, 4$) are arbitrary matrices with appropriate dimensions. Using the properties of the stochastic integral [41], we have

$$\begin{aligned}& \mathbf{E} \biggl\{ \bigl[x^{T} \bigl(t-\tau (t) \bigr) N_{1}+x^{T}_{\tau } \bigl(t-\tau (t) \bigr) N_{2} \bigr] \int _{t-\tau (t) }^{t} \bigl(Hx(s)+H_{\tau }x \bigl(s- \tau (s) \bigr)+E_{2}h(s) \bigr)\,\mathrm{d}B(s) \biggr\} =0, \\& \mathbf{E} \biggl\{ \bigl[x^{T}(t-\tau ) N_{3}+x_{\tau }(t- \tau ) N^{T}_{4} \bigr] \int _{t-\tau }^{t-\tau (t)} \bigl(Hx(s)+H_{\tau }x \bigl(s- \tau (s) \bigr)+E_{2}h(s) \bigr)\,\mathrm{d}B(s) \biggr\} =0. \end{aligned}$$

Adding the left-hand sides of (13) and (14) onto $\mathcal{L}V (x(t), t)$, (12) is transformed to

15 $$ \mathbf{E}\,\mathrm{d}V \bigl(x(t),t \bigr)=\mathbf{E}\mathcal{L} \tilde{V} \bigl(x(t),t \bigr)\,\mathrm{d}t, $$

where

16 $$\begin{aligned} &\mathcal{L}\tilde{V} \bigl(x(t),t \bigr) \\ &\quad = \mathcal{L}V \bigl(x(t),t \bigr)+ 2 \bigl[x^{T} \bigl(t-\tau (t) \bigr) N_{1}+x^{T}_{\tau } \bigl(t-\tau (t) \bigr) N_{2} \bigr] \biggl[x(t) \\ &\quad \quad {}-Cx \bigl(t-\tau (t) \bigr)-x \bigl(t-\tau (t) \bigr)+Cx_{\tau } \bigl(t-\tau (t) \bigr)- \int _{t-\tau (t) }^{t} h_{1}(s)\,\mathrm{d}s \biggr] \\ &\quad \quad {} +2 \bigl[x^{T}(t-\tau ) N_{3}+x^{T}_{\tau }(t- \tau ) N_{4} \bigr] \\ &\quad \quad {}\times \biggl[ x \bigl(t-\tau (t) \bigr)-Cx_{\tau } \bigl(t-\tau (t) \bigr)-x(t-\tau )+Cx_{\tau }(t-\tau )- \int _{t- \tau }^{t-\tau (t)}h_{1}(s)\,\mathrm{d}s \biggr]. \end{aligned}$$

For $\dot{\tau } (t)\leq d$, we have

17 $$\begin{aligned} &\mathcal{L}\tilde{V} \bigl(x(t),t \bigr) \\ &\quad = \mathcal{L}V \bigl(x(t),t \bigr)+ 2 \bigl[x^{T} \bigl(t-\tau (t) \bigr) N_{1}+x^{T}_{\tau } \bigl(t-\tau (t) \bigr) N_{2} \bigr] \biggl[x(t) \\ &\quad \quad {}-Cx \bigl(t-\tau (t) \bigr)-x \bigl(t-\tau (t) \bigr)+Cx_{\tau } \bigl(t-\tau (t) \bigr)- \int _{t-\tau (t) }^{t} z(s)\,\mathrm{d}s \biggr] \\ &\quad \quad {} +2 \bigl[x^{T}(t-\tau ) N_{3}+x^{T}_{\tau }(t- \tau ) N_{4} \bigr] \\ &\quad \quad {}\times \biggl[ x \bigl(t-\tau (t) \bigr)-Cx_{\tau } \bigl(t-\tau (t) \bigr)-x(t-\tau )+Cx_{\tau }(t-\tau ) - \int _{t- \tau }^{t-\tau (t)}h_{1}(s)\,\mathrm{d}s \biggr] \\ &\quad \leq \biggl\{ 2 \bigl[x(t)-Cx \bigl(t-\tau (t) \bigr) \bigr]^{T} P \bigl[Ax(t)+A_{\tau }x \bigl(t-\tau (t) \bigr) +f \bigl(x(t),x \bigl(t-\tau (t) \bigr) \bigr) \\ &\quad \quad {}+E_{1}h(t) \bigr] + \bigl[Hx(t)+H_{\tau }x \bigl(t-\tau (t) \bigr)+E_{2}h(t) \bigr]^{T} P \\ &\quad \quad {}\times \bigl[Hx(t)+H_{\tau }x \bigl(t-\tau (t) \bigr)+E_{2}h(t) \bigr]+x^{T}(t) W_{1} x(t) \\ &\quad \quad {}-(1-d)x^{T} \bigl(t-\tau (t) \bigr) W_{1} x \bigl(t-\tau (t) \bigr)+x^{T}(t) W_{2} x(t) \\ &\quad \quad {}-x^{T}(t-\tau ) W_{2} x(t-\tau )+\tau h_{1}^{T}(t) R h_{1}(t)- \int ^{t-\tau (t)}_{t-\tau }h_{1}^{T}(s)Rh_{1}(s) \,\mathrm{d}s \\ &\quad \quad {}- \int ^{t}_{t-\tau (t)}h_{1}^{T}(s)Rh_{1}(s) \,\mathrm{d}s \biggr\} + 2 \bigl[x^{T} \bigl(t-\tau (t) \bigr) N_{1}+x_{\tau }^{T} \bigl(t-\tau (t) \bigr) N_{2} \bigr] \\ &\quad \quad {}\times \biggl[x(t)-Cx \bigl(t-\tau (t) \bigr)-x \bigl(t-\tau (t) \bigr)+Cx_{\tau } \bigl(t-\tau (t) \bigr)- \int _{t-\tau (t) }^{t} h_{1}(s)\,\mathrm{d}s \biggr] \\ &\quad \quad {} +2 \bigl[x^{T}(t-\tau ) N_{3}+x_{\tau }^{T}(t- \tau ) N_{4} \bigr] \\ &\quad \quad {}\times \biggl[ x \bigl(t-\tau (t) \bigr)- Cx_{\tau } \bigl(t-\tau (t) \bigr)-x(t-\tau )+Cx_{\tau }(t-\tau )- \int _{t-\tau }^{t-\tau (t)}h_{1}(s)\,\mathrm{d}s \biggr]. \end{aligned}$$

On the other hand, by using the one-sided Lipschitz (1) and the quadratically inner-bounded condition (2), we obtain the following inequalities:

18 $$\begin{aligned}& \alpha _{1}x^{T}(t)x(t)+\alpha _{2}x^{T} \bigl(t-\tau (t) \bigr)x \bigl(t-\tau (t) \bigr)-x^{T}(t)f \bigl(x(t),x \bigl(t-\tau (t) \bigr) \bigr)\geq 0, \end{aligned}$$

19 $$\begin{aligned}& \beta _{1}x^{T}(t)x(t)+ \beta _{2}x^{T} \bigl(t-\tau (t) \bigr)x \bigl(t-\tau (t) \bigr)-f \bigl(x(t),x \bigl(t-\tau (t) \bigr) \bigr)^{T}f \bigl(x(t),x \bigl(t-\tau (t) \bigr) \bigr) \\& \quad {}+\gamma x^{T}(t)f \bigl(x(t),x \bigl(t-\tau (t) \bigr) \bigr)\geq 0. \end{aligned}$$

Using the S-procedure in (17), we obtain that $\mathcal{L}\tilde{V}(x(t),t)<0$ is satisfied if there exist positive scalars $\varepsilon _{1}$, $\varepsilon _{2}$, $\varepsilon _{3}$ satisfying

20 $$\begin{aligned} &\mathcal{L}\tilde{V} \bigl(x(t),t \bigr)+ \varepsilon _{1} \bigl[G_{1} x(t)+G_{2} x \bigl(t- \tau (t) \bigr) \bigr]^{T} \bigl[G_{1} x(t)+G_{2} x \bigl(t-\tau (t) \bigr) \bigr]-\varepsilon _{1}h(t)^{T}h(t) \\ &\quad {}+ \varepsilon _{2}\alpha _{1}x^{T}(t)x(t)+ \varepsilon _{2}\alpha _{2}x^{T} \bigl(t-\tau (t) \bigr)x \bigl(t-\tau (t) \bigr)-\varepsilon _{2}x^{T}(t)f \bigl(x(t),x \bigl(t-\tau (t) \bigr) \bigr) \\ &\quad {}+ \varepsilon _{3}\beta _{1}x^{T}(t)x(t)+ \varepsilon _{3}\beta _{2}x^{T} \bigl(t-\tau (t) \bigr)x \bigl(t-\tau (t) \bigr)-\varepsilon _{3}f \bigl(x(t),x \bigl(t- \tau (t) \bigr) \bigr)^{T} \\ &\quad {} \times f \bigl(x(t),x \bigl(t-\tau (t) \bigr) \bigr) +\varepsilon _{3} \gamma x^{T}(t)f \bigl(x(t),x \bigl(t-\tau (t) \bigr) \bigr)< 0. \end{aligned}$$

Moreover, the following formula holds for any positive definite matrix $M_{1} (M_{2},\ldots ,M_{5})$ of appropriate dimensions:

21 $$ \tau x^{T}(t)M_{1}x(t)- \int _{t-\tau }^{t} x^{T}(t)M_{1}x(t) \,\mathrm{d}s=0. $$

We can decompose the integration interval $[t-\tau , t]$ into two subintervals that are $[t-\tau , t-\tau (t)]$ and $[t-\tau (t),t]$, and let

$$\begin{aligned}& \xi ^{T}(t) = \bigl[x^{T}(t) x^{T} \bigl(t-\tau (t) \bigr) x^{T}(t-\tau ) x_{\tau }^{T} \bigl(t-\tau (t) \bigr) x_{\tau }^{T}(t-\tau ) f^{T} \bigl(x(t),x \bigl(t-\tau (t) \bigr) \bigr) h^{T}(t) \bigr], \\& \eta ^{T}(t,s) = \bigl[x^{T}(t) x^{T} \bigl(t- \tau (t) \bigr) x^{T}(t-\tau ) x_{\tau }^{T} \bigl(t- \tau (t) \bigr) x_{\tau }^{T}(t-\tau ) h_{1}^{T}(s) \bigr]. \end{aligned}$$

Combining the above formula (21) and rearranging (20), we have the following inequality:

22 $$\begin{aligned}& \xi ^{T}(t)\varOmega \xi (t)+\tau h_{1}^{T}(t)Rh_{1}(t) \\& \quad {}+ \int _{t-\tau }^{t-\tau (t)}\eta ^{T}(t,s)\varLambda \eta (t,s)\,\mathrm{d}s+ \int _{t-\tau (t)}^{t}\eta ^{T}(t,s)\varPi \eta (t,s) \,\mathrm{d}s< 0, \end{aligned}$$

where

23 $$\begin{aligned}& \varOmega = \begin{bmatrix} \varOmega _{11}& \varOmega _{12} & 0& N^{T}_{2}& 0& \varOmega _{16}&PE_{1}+H^{T}PE_{2} \\ \ast & \varOmega _{22}& N^{T}_{3}&\varOmega _{24}& N^{T}_{4}& -C^{T}P&-C^{T}PE_{1}+H_{\tau }^{T}PE_{2}\\ \ast & \ast & \varOmega _{33}& \varOmega _{34}& \varOmega _{35}& 0&0\\ \ast & \ast & \ast & \varOmega _{44}&\varOmega _{45}&0&0\\ \ast & \ast & \ast & \ast & \varOmega _{55}& 0&0\\ \ast & \ast & \ast & \ast & \ast & -\varepsilon _{3} I&0\\ \ast & \ast & \ast & \ast & \ast &\ast &E_{2}^{T}PE_{2} -\varepsilon _{1} I \end{bmatrix} < 0, \\& \varLambda = \begin{bmatrix} -M_{1}& 0 & 0& 0& 0& 0 \\ \ast & -M_{2}& 0 & 0& 0& 0\\ \ast & \ast & -M_{3}& 0 & 0& -N_{3}\\ \ast & \ast & \ast & -M_{4}& 0 & 0\\ \ast & \ast & \ast & \ast & -M_{5}&-N_{4}\\ \ast & \ast & \ast & \ast & \ast & -R \end{bmatrix} < 0, \\& \varPi = \begin{bmatrix} -M_{1}& 0 & 0& 0& 0& 0 \\ \ast & -M_{2}& 0 & 0& 0& -N_{1}\\ \ast & \ast & -M_{3}& 0 & 0& 0\\ \ast & \ast & \ast & -M_{4}& 0 & -N_{2}\\ \ast & \ast & \ast & \ast & -M_{5}& 0 \\ \ast & \ast & \ast & \ast & \ast & -R \end{bmatrix} < 0. \\& \varOmega _{11}=PA+A^{T}P+H^{T}PH+W_{1}+W_{2}+ \varepsilon _{1}G_{1}^{T}G_{1}+\tau M_{1} +\varepsilon _{2}\alpha _{1}I+\varepsilon _{3}\beta _{1}I, \\& \varOmega _{12}=PA_{\tau }-A^{T}PC+H^{T}PH_{\tau }+N_{1}^{T}+\varepsilon _{1}G_{1}^{T}G_{2}, \\& \varOmega _{16}= P-\frac{1}{2}\varepsilon _{2}I+\frac{1}{2}\varepsilon _{3}\gamma I, \\& \varOmega _{22}=-C^{T}PA_{\tau }-A_{\tau }^{T}PC+H_{\tau }^{T}PH_{\tau }-(1-d)W_{1}-N_{1}(C+I)-(C+I)^{T}N_{1}^{T} \\& \hphantom{\varOmega _{22}=-}{}+\varepsilon _{1}G_{2}^{T}G_{2}+\tau M_{2}+\varepsilon _{2}\alpha _{2}I+\varepsilon _{3}\beta _{2}I, \\& \varOmega _{24}=N_{1}C-C^{T}N_{2}^{T}-N_{2}^{T}, \\& \varOmega _{33}= -W_{2}+\tau M_{3}-N_{3}-N_{3}^{T}, \\& \varOmega _{34}= -N_{3}C, \\& \varOmega _{35}=N_{3}C-N_{4}^{T}, \\& \varOmega _{44}= N_{2}C+C^{T} N_{2}^{T}+ \tau M_{4}, \\& \varOmega _{45}= -C^{T} N_{4}^{T}, \\& \varOmega _{55}= N_{4}C+C^{T} N_{4}^{T}+ \tau M_{5}. \end{aligned}$$

Utilizing the Schur complement Lemma 2.1, (23) is equivalent to the following LMI:

24 $$\begin{aligned}& \bar{\varOmega }= \begin{bmatrix} \bar{\varOmega }_{11}& \bar{\varOmega }_{12}& 0 & N_{2}^{T}& 0& \bar{\varOmega }_{16}& \bar{\varOmega }_{17} &A^{T}&\bar{M_{1}} \\ \ast & \bar{\varOmega }_{22}& N_{3}^{T}&\bar{\varOmega }_{24} &N_{4}^{T}& -C^{T}P& \bar{\varOmega }_{27} &A^{T}_{\tau }&\bar{M_{2}}\\ \ast & \ast & \bar{\varOmega }_{33}& \bar{\varOmega }_{34}& \bar{\varOmega }_{35}& 0&0&0&\bar{M_{3}}\\ \ast & \ast & \ast & \bar{\varOmega }_{44}& \bar{\varOmega }_{45}&0&0&0&\bar{M_{4}}\\ \ast & \ast & \ast & \ast &\bar{\varOmega }_{55}& 0&0&0&\bar{M_{5}}\\ \ast & \ast & \ast & \ast & \ast & -\varepsilon _{3} I&0&I&0\\ \ast & \ast & \ast & \ast & \ast &\ast & \bar{\varOmega }_{77} &E_{1}^{T}&0\\ \ast & \ast & \ast & \ast & \ast &\ast &\ast &-\tau ^{-1}R^{-1}&0\\ \ast & \ast & \ast & \ast & \ast &\ast &\ast &\ast &-\bar{M} \end{bmatrix}< 0, \\& \bar{ \varOmega }_{11}= PA+A^{T}P+H^{T}PH+W_{1}+W_{2}+ \varepsilon _{1}G_{1}^{T}G_{1} + \varepsilon _{2}\alpha _{1}I+\varepsilon _{3} \beta _{1}I, \\& \bar{\varOmega }_{12}= PA_{\tau }-A^{T}PC+H^{T}PH_{\tau } +N_{1}^{T}+\varepsilon _{1}G_{1}^{T}G_{2}, \\& \bar{\varOmega }_{16}= P-\frac{1}{2}\varepsilon _{2}I+ \frac{1}{2}\varepsilon _{3}\gamma I,\qquad \bar{\varOmega }_{17}=PE_{1}+H^{T}PE_{2}, \\& \bar{\varOmega }_{22}= -C^{T}PA_{\tau }-A_{\tau }^{T}PC+H_{\tau }^{T}PH_{\tau }-(1-d)W_{1} \\& \hphantom{\bar{\varOmega }_{22}=}{}-N_{1}(C+I)-(C+I)^{T}N_{1}^{T}+ \varepsilon _{1}G_{2}^{T}G_{2}+ \varepsilon _{2}\alpha _{2}I+\varepsilon _{3} \beta _{2}I, \\& \bar{\varOmega }_{24}= N_{1}C-C^{T}N_{2}^{T}-N_{2}^{T}, \qquad \bar{\varOmega }_{27}=-C^{T}PE_{1}+H_{\tau }^{T}PE_{2}, \\& \bar{\varOmega }_{33}= -W_{2} -N_{3}-N_{3}^{T} \qquad \bar{\varOmega }_{34}= -N_{3}C,\qquad \bar{\varOmega }_{35}=N_{3}C-N_{4}^{T}, \\& \bar{\varOmega }_{44}= N_{2}C+C^{T} N_{2}^{T},\qquad \bar{\varOmega }_{45}= -C^{T} N_{4}^{T},\qquad \bar{\varOmega }_{55}= N_{4}C+C^{T} N_{4}^{T}, \\& \bar{\varOmega }_{77}= E_{2}^{T}PE_{2} - \varepsilon _{1} I,\qquad \bar{ M}= \operatorname{diag} \bigl\{ \tau ^{-1}M_{1}, \tau ^{-1}M_{2},\tau ^{-1}M_{3},\tau ^{-1}M_{4},\tau ^{-1}M_{5} \bigr\} , \\& \bar{ M}_{1}= [M_{1},0,0,0,0],\qquad \bar{ M}_{2}= [0,M_{2},0,0,0],\qquad \bar{ M}_{3}= [0,0,M_{3},0,0], \\& \bar{ M}_{4}= [0,0,0,M_{4},0],\qquad \bar{ M}_{5}= [0,0,0,0,M_{5}]. \end{aligned}$$

Pre-and post-multiplying (24) by $\operatorname{diag}\{I, I, I, I, I, I,I,R, I\}$, we obtain LMI (7). Combining with $\varLambda <0$ and $\varPi <0$, we find that $\mathbf{E}\mathcal{L}\tilde{V} (\xi (t), t)<0$, i.e., it guarantees the asymptotic stability of system (6) in the mean square. □

If the uncertain parameters $\Delta A(t)$, $\Delta A_{\tau }(t)$, $\Delta H(t)$, and $\Delta H_{\tau }(t)$ in system (6) are equal to zero, the system is simplified to the following deterministic stochastic system:

25 $$ \textstyle\begin{cases} \mathrm{d} [x(t)-Cx (t-\tau (t) ) ] \\ \quad = [Ax(t)+A_{\tau }x (t-\tau (t) )+f(x(t),x (t-\tau (t) )+Uu(t) ]\,\mathrm{d}t \\ \quad \quad {} + [Hx(t)+H_{\tau }x (t-\tau (t) ) ] \,\mathrm{d}B(t), \\ x(t)= \phi (t),\quad t\in [-\tau ,0]. \end{cases} $$

The following conclusion of the robust asymptotic stability is obtained by Theorem 4.1 for the deterministic stochastic system (25).

### Corollary 4.1

*Consider the stochastic time*-*delay system* (25). *The nonlinear function* $f(x(t),x(t-\tau (t)))$ *satisfies* (1) *and* (2). *For given scalars* *τ* *and* *d*, *if there exist matrices* $P>0$, $W_{1}>0$, $W_{2}>0$, $R>0$, $M_{i}>0$ ($i=1,\ldots ,5$), *and matrices* $N_{j}$ ($j=1,\ldots, 4$) *of appropriate dimensions and positive scalars* $\varepsilon _{1}$, $\varepsilon _{2}$, $\varepsilon _{3}$ *satisfying the following LMIs*:

26 $$ \begin{bmatrix} \varOmega _{11}& \varOmega _{12}& 0 & N_{2}^{T}& 0& \varOmega _{16}&A^{T}R&\bar{M}_{1} \\ \ast & \varOmega _{22}& N_{3}^{T}&\varOmega _{24} &N_{4}^{T}& -C^{T}P&A^{T}_{\tau }R&\bar{M}_{2}\\ \ast & \ast & \varOmega _{33}& -N_{3}C& \varOmega _{35}& 0&0&\bar{M}_{3}\\ \ast & \ast & \ast & \varOmega _{44}& -C^{T}N_{4}^{T}&0&0&\bar{M}_{4}\\ \ast & \ast & \ast & \ast &\varOmega _{55}& 0&0&\bar{M}_{5}\\ \ast & \ast & \ast & \ast & \ast & -\varepsilon _{3} I&R&0\\ \ast & \ast & \ast & \ast & \ast &\ast &-\tau ^{-1}R&0\\ \ast & \ast & \ast & \ast & \ast &\ast &\ast &-\bar{M} \end{bmatrix} < 0, $$

*where*

$$\begin{aligned}& \varOmega _{11}=PA+A^{T}P+H^{T}PH+W_{1}+W_{2} +\varepsilon _{2}\alpha _{1}I+\varepsilon _{3} \beta _{1}I, \\& \varOmega _{12}= PA_{\tau }-A^{T}PC+H^{T}PH_{\tau } +N_{1}^{T},\qquad \varOmega _{16}= P- \frac{1}{2} \varepsilon _{2}I+\frac{1}{2}\varepsilon _{3}\gamma I, \\& \varOmega _{22}= -C^{T}PA_{\tau }-A_{\tau }^{T}PC+H_{\tau }^{T}PH_{\tau }-(1-d)W_{1} \\& \hphantom{\varOmega _{22}=}{}-N_{1}(C+I)-(C+I)^{T}N_{1}^{T}+ \varepsilon _{2}\alpha _{2}I+\varepsilon _{3} \beta _{2}I, \\& \varOmega _{24}= N_{1}C-C^{T}N_{2}^{T}-N_{2}^{T}, \qquad \varOmega _{33}= -W_{2} -N_{3}-N_{3}^{T}, \\& \varOmega _{35}= N_{3}C-N_{4}^{T},\qquad \varOmega _{44}= N_{2}C+C^{T} N_{2}^{T}, \qquad \varOmega _{55}= N_{4}C+C^{T} N_{4}^{T}, \\& \bar{ M}= \operatorname{diag} \bigl\{ \tau ^{-1}M_{1},\tau ^{-1}M_{2},\tau ^{-1}M_{3},\tau ^{-1}M_{4},\tau ^{-1}M_{5} \bigr\} , \\& \bar{ M}_{1}= [M_{1},0,0,0,0],\qquad \bar{ M}_{2}=[0,M_{2},0,0,0],\qquad \bar{ M}_{3}= [0,0,M_{3},0,0], \\& \bar{ M}_{4}= [0,0,0,M_{4},0],\qquad \bar{ M}_{5}= [0,0,0,0,M_{5}], \end{aligned}$$

27 $$ \varLambda = \begin{bmatrix} -M_{1}& 0 & 0& 0& 0& 0 \\ \ast & -M_{2}& 0 & 0& 0& 0\\ \ast & \ast & -M_{3}& 0 & 0& -N_{3}\\ \ast & \ast & \ast & -M_{4}& 0 & 0\\ \ast & \ast & \ast & \ast & -M_{5}&-N_{4}\\ \ast & \ast & \ast & \ast & \ast & -R \end{bmatrix} < 0, $$

28 $$ \varPi = \begin{bmatrix} -M_{1}& 0 & 0& 0& 0& 0 \\ \ast & -M_{2}& 0 & 0& 0& -N_{1}\\ \ast & \ast & -M_{3}& 0 & 0& 0\\ \ast & \ast & \ast & -M_{4}& 0 & -N_{2}\\ \ast & \ast & \ast & \ast & -M_{5}& 0 \\ \ast & \ast & \ast & \ast & \ast & -R \end{bmatrix} < 0, $$

*the null solution of the stochastic time*-*delay system* (25) *is asymptotically stable in the mean square*.

## Non-fragile robust state feedback controller design

In this section, we consider the design of a non-fragile state-feedback controller:

29 $$ u(t)=K(t)x(t)= \bigl(K+\Delta K(t) \bigr)x(t), $$

guaranteeing the robust stability for the closed-loop system (3). *K* is the controller gain, and $\Delta K(t)$ represents the gain perturbations with the following assumption:

30 $$ \Delta K(t)=E_{3}F(t)G_{3}, $$

where $E_{3}$ and $G_{3}$ are given real constant matrices with appropriate dimensions.

### Theorem 5.1

*Consider the stochastic time*-*delay system* (6). *The nonlinear function* $f(x(t),x(t-\tau (t)))$ *satisfies* (1) *and* (2). *For given scalars* *τ* *and* *d*, *if there exist matrices* $P>0$, $W_{1}>0$, $W_{2}>0$, $R>0$, $M_{i}>0$ ($i=1,\ldots, 5$), *and matrices* $N_{j}$ ($j=1,\ldots, 4$) *of appropriate dimensions and positive scalars* $\varepsilon _{1}$, $\varepsilon _{2}$, $\varepsilon _{3}$, $\varepsilon _{4}$, *and* *σ* *satisfying the following LMIs*:

31 $$\begin{aligned}& \left [ \textstyle\begin{array}{@{}c@{\quad}c@{\quad}c@{\quad}c@{\quad}c@{\quad}c@{\quad}c@{\quad}c@{\quad}c@{\quad}c@{\quad}c@{\quad}@{}} \varOmega_{11} & \varOmega _{12} & 0 & N_{2}^{T} & 0 & \varOmega_{16} & \varOmega _{17} & A^{T}R & \bar{M_{1}} & J_{1} & 0 \\ \ast & \varOmega _{22} & N_{3}^{T} & \varOmega _{24} & N_{4}^{T} & -C^{T}P & \varOmega _{27} & A^{T}_{\tau }R & \bar{M_{2}} & 0 & 0 \\ \ast & \ast & \varOmega _{33} & \varOmega _{34} & \varOmega _{35} & 0 & 0 & 0 & \bar{M_{3}} & 0 & 0 \\ \ast & \ast & \ast & \varOmega _{44} & \varOmega _{45} & 0 & 0 & 0 & \bar{M_{4}} & 0 & 0 \\ \ast & \ast & \ast & \ast & \varOmega _{55} & 0 & 0 & 0 & \bar{M_{5}} & 0 & 0 \\ \ast & \ast & \ast & \ast & \ast & -\varepsilon _{3} I & 0 & R & 0 & 0 & 0 \\ \ast & \ast & \ast & \ast & \ast & \ast & \varOmega _{77} & E_{1}^{T}R & 0 & 0 & 0 \\ \ast & \ast & \ast & \ast & \ast & \ast & \ast & -\tau ^{-1}R & 0 & 0 & L_{1} \\ \ast & \ast & \ast & \ast & \ast & \ast & \ast & \ast & -\bar{M} & 0 & 0 \\ \ast & \ast & \ast & \ast & \ast & \ast & \ast & \ast & \ast & -J & 0 \\ \ast & \ast & \ast & \ast & \ast & \ast & \ast & \ast & \ast & \ast & -L \end{array}\displaystyle \right ] < 0, \end{aligned}$$

32 $$\begin{aligned}& \varLambda = \begin{bmatrix} -M_{1}& 0 & 0& 0& 0& 0 \\ \ast & -M_{2}& 0 & 0& 0& 0\\ \ast & \ast & -M_{3}& 0 & 0& -N_{3}\\ \ast & \ast & \ast & -M_{4}& 0 & 0\\ \ast & \ast & \ast & \ast & -M_{5}& -N_{4}\\ \ast & \ast & \ast & \ast & \ast & -R \end{bmatrix} < 0, \end{aligned}$$

33 $$\begin{aligned}& \varPi = \begin{bmatrix} -M_{1}& 0 & 0& 0& 0& 0 \\ \ast & -M_{2}& 0 & 0& 0& -N_{1}\\ \ast & \ast & -M_{3}& 0 & 0& 0\\ \ast & \ast & \ast & -M_{4}& 0 & -N_{2}\\ \ast & \ast & \ast & \ast & -M_{5}& 0 \\ \ast & \ast & \ast & \ast & \ast & -R \end{bmatrix} < 0, \end{aligned}$$

*where*

$$\begin{aligned}& \varOmega _{11}= PA+A^{T}P+H^{T}PH+W_{1}+W_{2}+ \varepsilon _{1}G_{1}^{T}G_{1} + \varepsilon _{2}\alpha _{1}I+\varepsilon _{3} \beta _{1}I+2\varepsilon _{4}G_{3}^{T}G_{3}, \\& \varOmega _{12}= PA_{\tau }-A^{T}PC+H^{T}PH_{\tau } +N_{1}^{T}+\varepsilon _{1}G_{1}^{T}G_{2}, \\& \varOmega _{16}= P-\frac{1}{2}\varepsilon _{2}I+ \frac{1}{2}\varepsilon _{3}\gamma I, \\& \varOmega _{17}= PE_{1}+H^{T}PE_{2}, \\& \varOmega _{22}= -C^{T}PA_{\tau }-A_{\tau }^{T}P C+H_{\tau }^{T}PH_{\tau }-(1-d)W_{1}-N_{1} C-C^{T}N_{1}^{T}-N_{1} \\& \hphantom{\varOmega _{22}=}{}-N_{1}^{T}+\varepsilon _{1}G_{2}^{T}G_{2}+ \varepsilon _{2}\alpha _{2}I+\varepsilon _{3} \beta _{2}I, \\& \varOmega _{24}= N_{1}C-C^{T}N_{2}^{T}-N_{2}^{T}, \\& \varOmega _{27}= -C^{T}PE_{1}+H_{\tau }^{T}PE_{2}, \qquad \varOmega _{33}=-W_{2}-N_{3}^{T}-N_{3}, \qquad \varOmega _{34}= -N_{3}C, \\& \varOmega _{35}= N_{3}C-N_{4}^{T},\qquad \varOmega _{44}= N_{2}C+C^{T} N_{2}^{T}, \qquad \varOmega _{45}=-C^{T} N_{4}^{T}, \\& \varOmega _{55}= N_{4}C+C^{T} N_{4}^{T}, \qquad \varOmega _{77}= E_{2}^{T}PE_{2} - \varepsilon _{1} I, \\& \bar{ M}= \operatorname{diag} \bigl\{ \tau ^{-1}M_{1},\tau ^{-1}M_{2},\tau ^{-1}M_{3},\tau ^{-1}M_{4},\tau ^{-1}M_{5} \bigr\} , \\& \bar{ M}_{1}= [M_{1},0,0,0,0],\qquad \bar{ M}_{2}= [0,M_{2},0,0,0],\qquad \bar{ M}_{3}= [0,0,M_{3},0,0], \\& \bar{ M}_{4}= [0,0,0,M_{4},0],\qquad \bar{ M}_{5}= [0,0,0,0,M_{5}],\qquad J= \operatorname{diag} \biggl\{ \frac{1}{2}\sigma I,\varepsilon _{4} I,\sigma I \biggr\} , \\& J_{1}= [PU,PUE_{3},PU],\qquad L= \operatorname{diag}\{ \varepsilon _{4} I,\sigma I\},\qquad L_{1}= [RUE_{3},RU], \end{aligned}$$

*the closed*-*loop systems are asymptotically stable in the mean square with the non*-*fragile state*-*feedback controller* $K=\sigma ^{-1}B^{T}P$.

### Proof

By using the controller $K=\sigma ^{-1}U^{T}P$ and letting

$$ h_{1}(t)= \bigl(A+U \bigl(K+\Delta K(t) \bigr) \bigr)x(t)+A_{\tau }x \bigl(t-\tau (t) \bigr)+f \bigl(x(t),x \bigl(t-\tau (t) \bigr) \bigr)+E_{1}h(t), $$

system (6) can be rewritten as follows:

34 $$ \textstyle\begin{cases} \mathrm{d} [x(t)-Cx (t-\tau (t) ) ] \\ \quad = h_{1}(t)\,\mathrm{d}t + [Hx(t)+H_{\tau }x (t-\tau (t) )+E_{2}h(t) ]\,\mathrm{d}B(t), \\ h(t)^{T}h(t) \leq [G_{1} x(t)+G_{2} x (t-\tau (t) ) ]^{T} [G_{1} x(t)+G_{2} x (t-\tau (t) ) ]. \end{cases} $$

Similar to Theorem 4.1, $\mathrm{E}\mathcal{L}\tilde{V}(\xi (t),t)<0$ is guaranteed by the following matrix inequality $\varOmega <0$:

$$ \varOmega = \begin{bmatrix} \varOmega _{11}& \varOmega _{12}& 0 & N_{2}^{T}& 0& \varOmega _{16}&\varOmega _{17}&\varOmega _{18}&\bar{M_{1}} \\ \ast & \varOmega _{22}& N_{3}^{T}&\varOmega _{24} & N_{4}^{T}&C^{T}P&\varOmega _{27}&A^{T}_{\tau }&\bar{M_{2}}\\ \ast & \ast & \varOmega _{33}& -N_{3}C& \varOmega _{35}& 0&0&0&\bar{M_{3}}\\ \ast & \ast & \ast & \varOmega _{44}& -C^{T}N_{4}^{T}&0&0&0&\bar{M_{4}}\\ \ast & \ast & \ast & \ast & \varOmega _{55}& 0&0&0&\bar{M_{5}}\\ \ast & \ast & \ast & \ast & \ast & -\varepsilon _{3} I&0&I&0\\ \ast & \ast & \ast & \ast & \ast &\ast &\varOmega _{77}&E_{1}^{T}&0\\ \ast & \ast & \ast & \ast & \ast &\ast &\ast &-\tau ^{-1}R^{-1}&0\\ \ast & \ast & \ast & \ast & \ast &\ast &\ast &\ast &-\bar{M} \end{bmatrix}, $$

where

$$\begin{aligned}& \varOmega _{11}= P(A+UK)+(A+UK)^{T}P+H^{T}PH+W_{1}+W_{2}+ \varepsilon _{1}G_{1}^{T}G_{1} \\& \hphantom{\varOmega _{11}= }{}+\varepsilon _{2}\alpha _{1}I+\varepsilon _{3} \beta _{1}I+PUE_{3}F(t)G_{3}+G_{3}^{T}F^{T}(t)E_{3}^{T}U^{T}P, \\& \varOmega _{12}= PA_{\tau }-A^{T}PC+H^{T}PH_{\tau } +N_{1}^{T}+\varepsilon _{1}G_{1}^{T}G_{2}, \\& \varOmega _{16}= P-\frac{1}{2}\varepsilon _{2}I+ \frac{1}{2}\varepsilon _{3}\gamma I, \\& \varOmega _{17}= PE_{1}+H^{T}PE_{2}, \\& \varOmega _{18}= A^{T}R+K^{T}U^{T}R+G_{3}F^{T}(t)E_{3}^{T}U^{T}R, \\& \varOmega _{22}= -C^{T}PA_{\tau }-A_{\tau }^{T}P C+H_{\tau }^{T}PH_{\tau }-(1-d)W_{1}-N_{1} C-C^{T}N_{1}^{T}-N_{1}-N_{1}^{T} \\& \hphantom{\varOmega _{22}=}{}+\varepsilon _{1}G_{2}^{T}G_{2}+ \varepsilon _{2}\alpha _{2}I+\varepsilon _{3} \beta _{2}I, \\& \varOmega _{24}= N_{1}C-C^{T}N_{2}^{T}-N_{2}^{T}, \\& \varOmega _{27}= -C^{T}PE_{1}+H_{\tau }^{T}PE_{2}, \qquad \varOmega _{33}= -W_{2}-N_{3}^{T}-N_{3}, \qquad \varOmega _{35}= N_{3}C-N_{4}^{T}, \\& \varOmega _{44}= N_{2}C+C^{T} N_{2}^{T}, \qquad \varOmega _{55}= N_{4}C+C^{T} N_{4}^{T},\qquad \varOmega _{77}= E_{2}^{T}PE_{2} - \varepsilon _{1} I \\& \bar{ M}= \operatorname{diag} \bigl\{ \tau ^{-1}M_{1},\tau ^{-1}M_{2},\tau ^{-1}M_{3},\tau ^{-1}M_{4},\tau ^{-1}M_{5} \bigr\} , \\& \bar{ M}_{1}= [M_{1},0,0,0,0],\qquad \bar{ M}_{2}= [0,M_{2},0,0,0],\qquad \bar{ M}_{3}= [0,0,M_{3},0,0], \\& \bar{ M}_{4}= [0,0,0,M_{4},0],\qquad \bar{ M}_{5}= [0,0,0,0,M_{5}]. \end{aligned}$$

According to condition (30), we have

$$ \varOmega =\hat{\varOmega }+\bar{E}F\bar{G}+\bar{G}^{T}F^{T} \bar{E}^{T}+\bar{K}\bar{R}+\bar{R}^{T}\bar{K}^{T}, $$

where

$$ \hat{\varOmega }= \begin{bmatrix} \hat{\varOmega }_{11} & \hat{\varOmega }_{12} & 0 & N_{2}^{T} & 0 & \hat{\varOmega }_{16} & \hat{\varOmega }_{17} & A^{T}R & \bar{M_{1}} \\ \ast & \hat{\varOmega }_{22} & N_{3}^{T} & \hat{\varOmega }_{24} & N_{4}^{T} & -C^{T}P & \hat{\varOmega }_{27} & A^{T}_{\tau } & \bar{M_{2}} \\ \ast & \ast & \hat{\varOmega }_{33} & -N_{3}C & N_{3}C-N_{4}^{T} & 0 & 0 & 0 & \bar{M_{3}} \\ \ast & \ast & \ast & \hat{\varOmega }_{44} & \hat{\varOmega }_{45} & 0 & 0 & 0 & \bar{M_{4}} \\ \ast & \ast & \ast & \ast & \hat{\varOmega }_{55} & 0 & 0 & 0 & \bar{M_{5}} \\ \ast & \ast & \ast & \ast & \ast & -\varepsilon _{3} I & 0 & I & 0 \\ \ast & \ast & \ast & \ast & \ast & \ast & \hat{\varOmega }_{77} & E_{1}^{T} & 0 \\ \ast & \ast & \ast & \ast & \ast & \ast & \ast & -\tau ^{-1}R^{-1} & 0 \\ \ast & \ast & \ast & \ast & \ast & \ast & \ast & \ast & -\bar{M} \end{bmatrix}, $$

where

$$\begin{aligned}& \hat{ \varOmega }_{11}= P(A+UK)+(A+UK)^{T}P+H^{T}PH+W_{1}+W_{2}+ \varepsilon _{1}G_{1}^{T}G_{1}+ \varepsilon _{2}\alpha _{1}I+\varepsilon _{3} \beta _{1}I, \\& \hat{\varOmega }_{12}= PA_{\tau }-A^{T}PC+H^{T}PH_{\tau } +N_{1}^{T}+\varepsilon _{1}G_{1}^{T}G_{2}, \\& \hat{\varOmega }_{16}= P-\frac{1}{2}\varepsilon _{2}I+ \frac{1}{2}\varepsilon _{3}\gamma I,\qquad \hat{\varOmega }_{17}=PE_{1}+H^{T}PE_{2}, \\& \hat{\varOmega }_{22}= -C^{T}PA_{\tau }-A_{\tau }^{T}P C+H_{\tau }^{T}PH_{\tau }-(1-d)W_{1}-N_{1} C-C^{T}N_{1}^{T}-N_{1}-N_{1}^{T} \\& \hphantom{\hat{\varOmega }_{22}= }{} +\varepsilon _{1}G_{2}^{T}G_{2}+ \varepsilon _{2}\alpha _{2}I+\varepsilon _{3} \beta _{2}I, \\& \hat{\varOmega }_{24}= N_{1}C-C^{T}N_{2}^{T}-N_{2}^{T}, \\& \hat{\varOmega }_{27}= -C^{T}PE_{1}+H_{\tau }^{T}PE_{2}, \\& \hat{ \varOmega }_{33}= -W_{2}-N_{3}^{T}-N_{3}, \\& \hat{\varOmega }_{44}= N_{2}C+C^{T} N_{2}^{T}, \\& \hat{\varOmega }_{45}= -C^{T} N_{4}^{T}, \\& \hat{\varOmega }_{55}= N_{4}C+C^{T} N_{4}^{T}, \\& \hat{\varOmega }_{77}= E_{2}^{T}PE_{2} -\varepsilon _{1} I, \\& \bar{ M}= \operatorname{diag} \bigl\{ \tau ^{-1}M_{1},\tau ^{-1}M_{2},\tau ^{-1}M_{3},\tau ^{-1}M_{4},\tau ^{-1}M_{5} \bigr\} ,\qquad \bar{ M}_{1}= [M_{1},0,0,0,0], \\& \bar{ M}_{2}= [0,M_{2},0,0,0],\qquad \bar{ M}_{3}= [0,0,M_{3},0,0], \\& \bar{ M}_{4}= [0,0,0,M_{4},0],\qquad \bar{ M}_{5}= [0,0,0,0,M_{5}]. \end{aligned}$$

Other notations are defined by (28), and $\bar{E}=\{(PUE_{3})_{1,1}, (RUE_{3})_{8,2}\}$ denotes a block matrix with appropriate dimensions whose all nonzero blocks are the $(1,1)$-block $PUE_{3}$, the $(8,2)$-block $RUE_{3}$, and all other blocks are zero matrices. Similarly, $\bar{G}=\{(G_{3})_{1,1}, (G_{3})_{2,1}\}$, $\bar{K}=\{(K^{T})_{1,1}\}$, $\bar{R}=\{(U^{T}R)_{1,8}\}$. According to Lemma 2.2, for any scalars $\varepsilon _{4}>0$ and $\sigma >0$, we have

35 $$ \varOmega < \hat{\varOmega }+\varepsilon _{4}^{-1} \bar{E}\bar{E}^{T}+\varepsilon _{4}\bar{G}^{T} \bar{G}+\sigma \bar{K}\bar{K}^{T}+\sigma ^{-1} \bar{R}^{T}\bar{R}. $$

Let $K=\sigma ^{-1}U^{T}P$, using the Schur Lemma 2.1, we arrive at

$$ \hat{\varOmega }+\varepsilon _{4}^{-1}\bar{E} \bar{E}^{T}+\varepsilon _{4}\bar{G}^{T}\bar{G}+ \sigma \bar{K}\bar{K}^{T}+\sigma ^{-1}\bar{R}^{T} \bar{R}< 0 $$

if LMI (31) is satisfied. □

## Two illustrative numerical examples

In order to illustrate the flexibility and reduced conservativeness of the proposed results, we present two numerical examples in this section.

### Example 6.1

[17] Consider the neutral stochastic time-delay nonlinear system (25) with

$$ A= \begin{bmatrix} -1.2& 0.1 \\ -0.1& -1 \end{bmatrix}, \qquad A_{\tau }= \begin{bmatrix} -0.6& 0.7 \\ -1& -0.8 \end{bmatrix}, $$

and the following nonlinear function that satisfies the one-sided Lipschitz (1) and the quadratically innerbounded conditions (2):

$$ f \bigl(x(t),x \bigl(t-\tau (t) \bigr) \bigr)= \begin{bmatrix} \frac{0.1}{sin^{2}(x_{2}-2)x_{1}(t-\tau (t))} \\ \frac{0.1}{sin^{1}(x_{1}-2)x_{2}(t-\tau (t))} \end{bmatrix}, $$

with $\alpha _{1}=0.5$, $\alpha _{2}=0.005$, $\gamma =5, \beta _{1}=-2.5$, $\beta _{2}=-0.015$ (see [17]). We consider the coefficient matrix *C* as follows:

$$ C= \begin{bmatrix} -0.1& 0.09 \\ -0.02& -1 \end{bmatrix}. $$

By Corollary 4.1, we can obtain the allowable upper bound of the time-delay $\tau =3.0345$ for the above systems, which is greater than the previous result (the allowable upper bound $\tau =2.2487$ in [17]). Comparing the allowable values of the systems, we see that our result are less conservative. Since our system considers neutral stochastic systems with derivatives of state time-delay, our results may have more extensive applications.

### Example 6.2

Consider the uncertain neutral stochastic nonlinear time-delay system (3) with

$$\begin{aligned}& A= \begin{bmatrix} -2.0& 0.0 \\ 0.0& -0.9 \end{bmatrix}, \qquad A_{\tau }= \begin{bmatrix} -1.0& 0.0 \\ -1.0& -1.0 \end{bmatrix}, \\& C= \begin{bmatrix} 0.05 &0 \\ 0.1 & 0.05 \end{bmatrix}, \qquad B= \begin{bmatrix} 1 \\ 2 \end{bmatrix}, \\& H= \begin{bmatrix} -0.3& 0.1 \\ 0.1& 0.2 \end{bmatrix}, \qquad H_{\tau }= \begin{bmatrix} -0.2& 0.2 \\ 0.3& -0.2 \end{bmatrix}, \\& E_{1}= \begin{bmatrix} 0.2& 0 \\ 0& 0.2 \end{bmatrix},\qquad E_{2}= \begin{bmatrix} 0.2& 0 \\ 0& 0.2 \end{bmatrix},\qquad E_{3}= \begin{bmatrix} 0.2& 0.2 \end{bmatrix}, \\& G_{1}= \begin{bmatrix} 0.2& 0 \\ 0& 0.2 \end{bmatrix}, \qquad G_{2}= \begin{bmatrix} 0.2& 0 \\ 0& 0.2 \end{bmatrix}, \qquad G_{3}= \begin{bmatrix} 0.2& 0 \\ 0& 0.2 \end{bmatrix}. \end{aligned}$$

Selecting the same nonlinear function $f(x(t), x(t-\tau (t)))$ as Example 6.1 and letting $d = 1.1$, we solve LMIs (31), (32), and (33) to obtain the allowable bound $\tau = 3.2106$. Hence, for any time-delay *τ* satisfying $0 < \tau \leq 3.2106$, there exists a non-fragile state-feedback controller such that the closed-loop systems are asymptotically stable in the mean square. For this example, if we choose the time-delay as $\tau = 2$, according to Theorem 5.1, we can obtain a set of solutions as follows:

$$\begin{aligned}& P= \begin{bmatrix} 4.4021 & -0.9004\\ -0.9004 & 0.8275 \end{bmatrix}, \\& W_{1}= \begin{bmatrix} 1.6119& -0.0127\\ -0.0127 & 2.6697 \end{bmatrix}, \qquad W_{2}= \begin{bmatrix} 28.4346 & -0.5875\\ -0.5875 & 28.2063 \end{bmatrix}, \\& R= \begin{bmatrix} 1.8078 & -0.6499\\ -0.6499 & 0.8340 \end{bmatrix}, \\& N_{1}= \begin{bmatrix} -0.0561 & -0.1719\\ -0.1719 & 0.0351 \end{bmatrix}, \qquad N_{2}= \begin{bmatrix} -0.0367 & -0.0039\\ -0.0039 & -0.0085 \end{bmatrix}, \\& N_{3}= \begin{bmatrix} -0.0114 & -0.1333\\ -0.1333 & 0.0285 \end{bmatrix}, \qquad N_{4}= \begin{bmatrix} -0.0190 & -0.0003\\ -0.0003 & -0.0034 \end{bmatrix}, \\& M_{1}= \begin{bmatrix} 9.9084 & -0.2109\\ -0.2109 & 10.0265 \end{bmatrix},\qquad M_{2}= \begin{bmatrix} 0.4494 & 0.0197\\ 0.0197 & 0.8863 \end{bmatrix}, \\& M_{3}= \begin{bmatrix} 10.1539 & -0.1770\\ -0.1770 & 10.2906 \end{bmatrix}, \qquad M_{4}= \begin{bmatrix} 0.0014 & 0.0004\\ 0.0004 & 0.0003 \end{bmatrix}, \\& M_{5}=1.0 \times 10^{-3} \begin{bmatrix} 0.4711 & 0.0875\\ 0.0875 & 0.0850 \end{bmatrix}, \\& \varepsilon _{1}=8.9466, \qquad \varepsilon _{2}= 1.0763 \times 10^{3},\qquad \varepsilon _{3}=255.2072, \qquad \varepsilon _{4}= 29.2135, \\& \sigma =38.2553, \end{aligned}$$

and the desired state-feedback matrix $K=\sigma ^{-1}B^{T}P=[0.0680 \ 0.0197]$.

## Conclusions

In this work, the robust stability has been investigated for neutral stochastic time-delay systems with disturbance, uncertainties, and one-sided Lipschitz nonlinearity. The parametric uncertainties are assumed to be time-varying and norm bounded. Firstly, the allowable upper bound of time-delay has been obtained, which is a less conservative result. Secondly, based on Lyapunov stability, a novel non-fragile state-feedback controller has been designed to guarantee the robust stability of the closed-loop systems. Finally, two numerical examples have been given to illustrate the effectiveness of the proposed control scheme.
